# When Appearance Comparisons Become Self-Disgust: The Protective Role of Self-Compassion in Disordered Eating

**DOI:** 10.62641/aep.2439

**Published:** 2026-10-15

**Authors:** Irene Sierra, Celia Ceballos-Munuera, Ángela Rosales-Becerra, Juan F. Rodríguez-Testal, Cristina Senín-Calderón

**Affiliations:** ^1^Department of Psychology, University of Cádiz, 11519 Puerto Real, Spain; ^2^Department of Personality, Evaluation and Psychological Treatments, University of Sevilla, 41018 Seville, Spain

**Keywords:** appearance-related social comparison, self-disgust, self-compassion, disordered eating, body image

## Abstract

**Background:**

Not everyone who compares their appearance with others develops eating-related difficulties. Understanding why some individuals are more vulnerable than others to the psychological consequences of appearance-related comparisons remains an important challenge. This study examined whether self-disgust helps explain the association between appearance-related social comparison and disordered eating and whether self-compassion weakens this pathway.

**Methods:**

A total of 812 young adults (53.8% women; mean age = 22.40 years, standard deviation [SD] = 3.53) completed measures of appearance-related social comparison (Physical Appearance Comparison Scale–Revised), self-disgust (Self-Disgust Scale), self-compassion (Self-Compassion Scale), and disordered eating (Eating Attitudes Test-26). A moderated mediation model (PROCESS Model 7) was tested controlling for sex.

**Results:**

The indirect association between appearance-related social comparison and disordered eating through self-disgust was significant, while the direct association remained significant. Self-compassion moderated the association between appearance-related social comparison and self-disgust (B = –1.31, *p* < 0.001), and the conditional indirect effect decreased from 0.99 at low self-compassion to 0.40 at high self-compassion. The mediator and outcome models explained 26.7% and 36.7% of the variance, respectively. The index of moderated mediation was significant (index = –0.42, 95% confidence interval [–0.69, –0.18]).

**Conclusions:**

Appearance-related comparisons may become harmful when perceived appearance discrepancies are internalized as self-disgust. More importantly, self-compassion appears to reduce the likelihood that appearance-related discrepancies are translated into self-disgust and eating-related difficulties. These findings suggest that vulnerability to disordered eating may depend not only on how individuals evaluate their appearance, but also on how they relate to themselves when confronted with perceived appearance inadequacies.

## Introduction

Disordered eating is a multifactorial phenomenon shaped by sociocultural, interpersonal, and intrapersonal influences [1, 2, 3, 4]. Among these, appearance-related social comparison has consistently emerged as one of the most robust correlates of disordered eating. Individuals who frequently compare their appearance with that of others tend to report greater body dissatisfaction, stronger internalization of appearance ideals, and higher levels of eating-related psychopathology [5, 6]. Yet, the strength of this association may depend on how perceived appearance discrepancies are interpreted and integrated into the self-concept.

One mechanism that may help explain the association between appearance-related social comparison and disordered eating is self-disgust. Self-disgust is a self-conscious emotion characterized by feelings of revulsion, rejection, and aversion directed toward the self [7, 8]. Whereas body dissatisfaction refers to discontent with particular physical attributes or aspects of one’s appearance (e.g., “I dislike my thighs”), self-disgust entails a broader and more visceral rejection of the self as unacceptable or repellent (e.g., “I detest who I am”). As such, it has increasingly been conceptualized as an extreme form of negative self-relating with transdiagnostic relevance across psychological disorders [9]. This distinction may be particularly relevant in appearance-related contexts, where perceived discrepancies from socially valued appearance standards may be internalized not merely as dissatisfaction with one’s body, but as rejection of the self, thereby providing a conceptual basis for the proposed mediating role of self-disgust.

Self-disgust has been linked to body dissatisfaction, body image disturbance, and eating pathology, and appears to contribute uniquely to appearance-related difficulties beyond broader indicators of emotional vulnerability [10]. Evidence from clinical samples indicates elevated levels of self-disgust among individuals with eating disorders compared with healthy controls, while emerging evidence suggests that self-disgust may contribute to the maintenance of negative body image through maladaptive cognitive and attentional processes [11, 12, 13]. Collectively, these findings point to self-disgust as a plausible mechanism through which appearance-related self-evaluations become translated into disordered eating. More broadly, they suggest that the way individuals relate to themselves in response to perceived appearance inadequacies may be central to understanding vulnerability to eating-related difficulties. Nevertheless, whether self-disgust explains the association between appearance-related social comparison and disordered eating remains largely unexplored.

If self-disgust represents one maladaptive way of responding to perceived appearance inadequacies, an important question is whether some individuals possess psychological resources that protect against this process. Self-compassion may represent one such factor. Defined as responding to personal inadequacies with kindness, understanding, and balanced awareness rather than self-criticism and self-condemnation [14], self-compassion has consistently been associated with lower levels of disordered eating and body image concern [15]. Evidence further suggests that these associations are partly explained by lower levels of shame, self-criticism, and other maladaptive forms of self-evaluation [16, 17]. From this perspective, self-compassion may not simply reduce eating-related difficulties directly but may alter the way appearance-based discrepancies are processed and integrated into the self. Individuals high in self-compassion may be less likely to interpret perceived appearance inadequacies as evidence of personal defectiveness and, consequently, less likely to respond with self-disgust. In turn, this may reduce vulnerability to disordered eating [15, 18].

Despite growing evidence linking appearance-related social comparison, self-disgust, self-compassion, and disordered eating, these constructs have rarely been examined within a single explanatory framework. To our knowledge, no previous study has investigated self-disgust as a mechanism linking appearance-related social comparison to disordered eating while simultaneously examining self-compassion as a protective factor capable of attenuating this pathway. Addressing this gap may contribute to a more nuanced understanding of how appearance-related experiences become translated into eating-related difficulties.

Building on this framework, the present study examined the role of self-disgust and self-compassion in the association between appearance-related social comparison and disordered eating.

Specifically, we hypothesized that:

- H1. Self-disgust would mediate the association between appearance-related social comparison and disordered eating.
- H2. Self-compassion would moderate the association between appearance-related social comparison and self-disgust, such that this association would be weaker among individuals with higher levels of self-compassion.
- H3. The indirect effect of appearance-related social comparison on disordered eating through self-disgust would be weaker at higher levels of self-compassion.

## Materials and Methods

### Participants and Procedure

Participants were Spanish young adults aged between 18 and 35 years. Demographic characteristics of the analytical sample are reported at the beginning of the Results section.

Participants were recruited using a structured non-probabilistic snowball sampling procedure. Undergraduate psychology students served as initial recruitment contacts and were asked to distribute the survey to approximately five eligible individuals from their personal networks. These participants were then invited to share the survey with other potential respondents. This procedure aimed to increase heterogeneity in the sample and facilitate recruitment from the general community.

The study received ethical approval from the Research Ethics Committee of the Andalusian Regional Government (reference number: PACS-2019) and was conducted in accordance with the ethical principles outlined in the Declaration of Helsinki [19]. Data collection was carried out between March and June 2024.

The survey was administered online via Google Forms. Participation was voluntary and anonymous, and all participants provided informed consent before completing the questionnaire. Cases with incomplete data on the study variables were excluded from the analyses.

### Measures

#### Physical Appearance Comparison Scale–Revised (PACS-R)

Physical Appearance Comparison Scale–Revised (PACS-R) [6]. Appearance-related social comparison was assessed using the Spanish version of the PACS-R [20]. The PACS-R is an 11-item self-report measure that assesses the frequency with which individuals compare their physical appearance with that of others in everyday situations. Responses are rated from 0 (never) to 4 (always). The mean of the 11 items was calculated, yielding a possible score range of 0–4, with higher scores indicating greater appearance-related social comparison. Internal consistency in the present sample was excellent (Cronbach’s α = 0.94).

#### Self-Disgust Scale (SDS)

Self-Disgust Scale (SDS) [7]. Self-disgust was assessed using a Spanish adaptation developed by the research team with permission from the original author. The measure was adapted into Spanish using a forward- and back-translation procedure conducted by two expert translators: a native Spanish speaker and a native English speaker from the United States. The procedure followed established recommendations for cross-cultural test adaptation [21]. Following translation, the preliminary Spanish version was pilot-tested with 50 psychology students, who were asked to indicate whether the items were understandable and clearly worded.

The original SDS contains 18 statements, comprising 12 substantive self-disgust items and six neutral filler items. Because the filler items are not included in the scoring of the scale, only the 12 substantive items were administered in the present study.

Preliminary psychometric analyses of the Spanish version were conducted using the same sample of 812 participants included in the present study. These analyses focused on the 10 items identified as factor-specific items in the rotated component solution reported by Overton *et al*. [7]: five items corresponding to Disgusting Self and five corresponding to Disgusting Ways. The two remaining substantive items were not retained because they were not assigned to either factor in the original solution and showed cross-loadings across the two components. For cross-validation purposes, the sample was randomly divided into two non-overlapping subsamples. An exploratory factor analysis (EFA) was conducted in the first subsample (n = 409), whereas a confirmatory factor analysis (CFA) was performed in the second subsample (n = 403). The subsamples were comparable in terms of sex distribution, χ^2^(1) = 0.02, *p* = 0.901, and age, *t*(809.37) = 0.71, *p* = 0.477.

The EFA was conducted using ordinary least squares extraction and oblimin rotation. Sampling adequacy was excellent (KMO = 0.929), and Bartlett’s test of sphericity was significant, χ^2^(45) = 2575.63, *p* < 0.001. A prespecified two-factor solution, corresponding to Disgusting Self and Disgusting Ways, accounted for 61.7% of the variance. The two-factor structure was subsequently examined in the second subsample using CFA with diagonally weighted least squares estimation. The model showed good global fit, Comparative Fit Index [CFI] = 0.997, Tucker–Lewis Index [TLI] = 0.996, Root Mean Square Error of Approximation [RMSEA] = 0.062, 90% CI [0.046, 0.079], and Standardized Root Mean Square Residual [SRMR] = 0.048.

Only the five-item Disgusting Self subscale was included in the present study because it assesses a global and visceral form of self-directed rejection and aversion, conceptually distinct from dissatisfaction with specific physical attributes, and is therefore consistent with the hypothesized mechanism linking appearance-related social comparison to disordered eating. Items were rated on a 7-point scale and summed, yielding a possible score range of 5–35, with higher scores indicating greater self-disgust. Internal consistency for the Disgusting Self subscale was high in the present sample (Cronbach’s α = 0.90). A full independent validation of the Spanish SDS has not yet been published.

#### The Self-Compassion Scale (SCS)

The Self-Compassion Scale (SCS) [22]. Self-compassion was assessed using the Spanish version of the 26-item SCS [23]. Responses are rated on a 5-point Likert scale ranging from 1 (almost never) to 5 (almost always). Although the appropriateness of combining the positively and negatively worded components into an overall score remains debated [24], the total score was calculated following the original scoring procedure [22] and psychometric evidence supporting its use [25]. Items assessing self-judgment, isolation, and over-identification were reverse coded, and the mean across all 26 items was calculated (possible range: 1–5), with higher scores indicating greater self-compassion. The total score was selected a priori because self-compassion was operationalized as an integrated multidimensional protective construct. Internal consistency in the present sample was excellent (Cronbach’s α = 0.91).

#### The Eating Attitudes Test-26 (EAT-26)

The Eating Attitudes Test-26 (EAT-26) [26]. Disordered eating attitudes and behaviours were assessed using the Spanish version of the EAT-26 [27]. The EAT-26 is a 26-item self-report measure designed to assess attitudes, concerns, and behaviours associated with eating-disorder symptomatology. Items are rated on a six-category response scale and recoded according to the standard EAT-26 scoring procedure. Item scores were summed (possible range: 0–78), with higher scores indicating greater disordered eating. The total score was analysed continuously and was not interpreted as a diagnostic classification. Internal consistency in the present sample was high (Cronbach’s α = 0.88).

### Data Analysis

Data analyses were conducted using IBM SPSS Statistics version 30 (IBM Corp., Armonk, NY, USA). Preliminary descriptive analyses were conducted. Sex differences were examined using independent-samples *t*-tests, with Welch corrections when homogeneity of variance was not supported. Cohen’s d was reported and interpreted using the conventional benchmarks of approximately 0.20, 0.50, and 0.80 for small, medium, and large effects, respectively [28].

Because several variables departed from normality, bivariate associations were examined using Spearman rank-order correlations. Multicollinearity was evaluated using variance inflation factors. Regression diagnostics included inspection of residual distributions, heteroscedasticity, and influential observations. Because heteroscedasticity was detected in the outcome model, HC3 heteroscedasticity-consistent standard errors and confidence intervals (CIs) are reported for the regression coefficients.

To test the study hypotheses, a moderated mediation analysis was conducted using Hayes’ PROCESS macro (version 5.0) [29]. Specifically, Model 7 was employed to examine whether self-disgust mediated the association between appearance-related social comparison and disordered eating and whether self-compassion moderated the relationship between appearance-related social comparison and self-disgust. Sex was included as a covariate in all analyses given the well-established sex differences reported in appearance-related social comparison, self-compassion, and disordered eating.

Indirect effects were estimated using 5000 percentile bootstrap samples. Evidence for moderated mediation was determined by examining the index of moderated mediation and its corresponding 95% bootstrap CI. Mediation and moderated mediation effects were considered statistically significant when the 95% bootstrap CIs did not include zero. Conditional indirect effects were estimated at low (16th percentile), moderate (50th percentile), and high (84th percentile) levels of self-compassion. Supplementary analyses treated sex as a moderator of the constituent pathways. Interaction terms tested whether sex moderated the first-stage PACS-R × SCS effect, the association between self-disgust and disordered eating, and the direct association between appearance-related social comparison and disordered eating. Between-sex differences in conditional indirect effects and indices of moderated mediation were evaluated using 5000 percentile bootstrap samples. All statistical tests were two-tailed, and statistical significance was set at *p* < 0.05.

## Results

### Preliminary Analyses

The analytical sample consisted of 812 Spanish young adults aged 18 to 35 years (mean [M] = 22.40, standard deviation [SD] = 3.53), including 375 men (46.2%) and 437 women (53.8%). Participants were predominantly from middle socioeconomic backgrounds, as indicated by the Four Factor Index of Social Status [30] (M = 40.17, SD = 17.62). Body mass index ranged from 15.43 to 48.44 kg/m^2^ (M = 23.42, SD = 4.33).

As shown in Table 1, women scored higher than men on appearance-related social comparison, Welch’s *t*(809.80) = −9.24, *p* < 0.001, d = 0.64; self-disgust, Welch’s *t*(808.72) = −2.10, *p* = 0.036, d = 0.15; and disordered eating, Welch’s *t*(798.62) = −3.95, *p* < 0.001, d = 0.27. Conversely, men reported higher levels of self-compassion, Welch’s *t*(807.08) = 3.87, *p* < 0.001, d = −0.27. According to Cohen’s benchmarks [28], the appearance-comparison difference was medium, whereas the remaining differences were small.

**Table 1.** **Descriptive statistics and sex differences across study variables**.

Variable	Men, M (SD)	Women, M (SD)	Welch *t*(df)	*p*	d
PACS-R	1.21 (0.82)	1.78 (0.94)	−9.24 (809.80)	<0.001	0.64
SDS-Disgusting Self	11.85 (7.08)	12.96 (7.93)	−2.10 (808.72)	0.036	0.15
SCS	3.11 (0.63)	2.93 (0.69)	3.87 (807.08)	<0.001	−0.27
EAT-26	6.51 (7.74)	9.00 (10.18)	−3.95 (798.62)	<0.001	0.27

Note. PACS-R, Physical Appearance Comparison Scale–Revised; SDS, Self-Disgust Scale; SCS, Self-Compassion Scale; EAT-26, Eating Attitudes Test–26; M, mean; SD, standard deviation; df, degrees of freedom; d, Cohen’s d. Positive d values indicate higher scores among women, whereas negative d values indicate higher scores among men.

Spearman correlation analyses revealed significant associations among all variables included in the proposed model (see Table 2). Appearance-related social comparison was positively associated with both self-disgust and disordered eating, while self-disgust was also positively related to disordered eating. In contrast, self-compassion showed negative associations with appearance-related social comparison, self-disgust, and disordered eating. No evidence of problematic multicollinearity was observed (Variance Inflation Factors [VIFs] = 1.11–1.38).

**Table 2.** **Descriptive statistics and Spearman correlations among study variables (n = 812)**.

Variable	M	SD	1	2	3	4
1. PACS-R	1.52	0.93	—			
2. SDS	12.45	7.56	0.43***	—		
3. SCS	3.02	0.67	−0.35***	−0.48***	—	
4. EAT-26	7.85	9.21	0.52***	0.46***	−0.33***	—

Note. PACS-R, Physical Appearance Comparison Scale–Revised; SDS, Self-Disgust Scale; SCS, Self-Compassion Scale; EAT-26, Eating Attitudes Test–26; M, mean; SD, standard deviation. PACS-R and SCS values are item means; SDS and EAT-26 values are summed scores. Spearman rank-order correlations are presented. ***p < 0.001.

### Moderated Mediation Analysis

Results of the moderated mediation analysis are presented in Table 3. The model predicting self-disgust explained 26.7% of the variance. The PACS-R × SCS interaction accounted for an additional 1.4% of the variance, △R^2^ = 0.014, F(1, 807) = 15.38, *p* < 0.001. Using HC3 inference, the interaction remained significant, B = −1.31, SEHC3 = 0.35, *p* < 0.001. Conditional effects indicated that the positive association between appearance-related social comparison and self-disgust was strongest at low self-compassion, B = 3.07, SE = 0.33, *p* < 0.001; weaker at the median, B = 2.22, SE = 0.28, *p* < 0.001; and weakest at high self-compassion, B = 1.26, SE = 0.40, *p* = 0.002. The covariate-adjusted model is shown in Fig. 1.

**Table 3.** **Moderated mediation model examining the association between appearance-related social comparison and disordered eating through self-disgust, conditional on self-compassion (n = 812)**.

**Panel A. Regression models**.						
Outcome	Predictor	B	SEHC3	*t*	*p*	95% CI
SDS (R^2^ = 0.267)	PACS-R	6.15	1.04	5.93	<0.001	[4.11, 8.18]
	SCS	−1.66	0.67	−2.47	0.014	[−2.98, −0.34]
	PACS-R × SCS	−1.31	0.35	−3.78	<0.001	[−1.99, −0.63]
	Sex	−0.90	0.48	−1.89	0.060	[−1.84, 0.04]
EAT-26 (R^2^ = 0.367)	PACS-R	4.53	0.37	12.32	<0.001	[3.81, 5.26]
	SDS	0.32	0.05	6.89	<0.001	[0.23, 0.41]
	Sex	−0.45	0.52	−0.87	0.383	[−1.46, 0.56]

**Table:** 

**Panel B. Conditional indirect effects of appearance-related social comparison on disordered eating through self-disgust**.			
Level of self-compassion	Indirect effect	BootSE	95% BootCI
Low (16th percentile)	0.99	0.19	[0.64, 1.39]
Moderate (50th percentile)	0.71	0.15	[0.44, 1.03]
High (84th percentile)	0.40	0.15	[0.13, 0.74]
Index of moderated mediation	−0.42	0.13	[−0.69, −0.18]

**Table:** 

**Panel C. Conditional effects of appearance-related social comparison on self-disgust**.					
Self-compassion	B	SEHC3	*t*	*p*	95% CI
Low (16th percentile)	3.07	0.33	9.41	<0.001	[2.43, 3.71]
Moderate (50th percentile)	2.22	0.28	7.93	<0.001	[1.67, 2.76]
High (84th percentile)	1.26	0.40	3.16	0.002	[0.48, 2.04]

Note. PACS-R, Physical Appearance Comparison Scale–Revised; SDS, Self-Disgust Scale; SCS, Self-Compassion Scale; EAT-26, Eating Attitudes Test–26; B, unstandardized regression coefficient; SE HC3, heteroscedasticity-consistent standard error (HC3); CI, confidence interval; BootSE, bootstrap standard error; BootCI, bootstrap confidence interval; R^2^, coefficient of determination. Sex was included as a covariate in all analyses. Regression coefficients use HC3 heteroscedasticity-consistent standard errors. Indirect effects were estimated using 5000 percentile bootstrap samples. Bootstrap confidence intervals excluding zero indicate statistical significance.

**Fig. 1.**
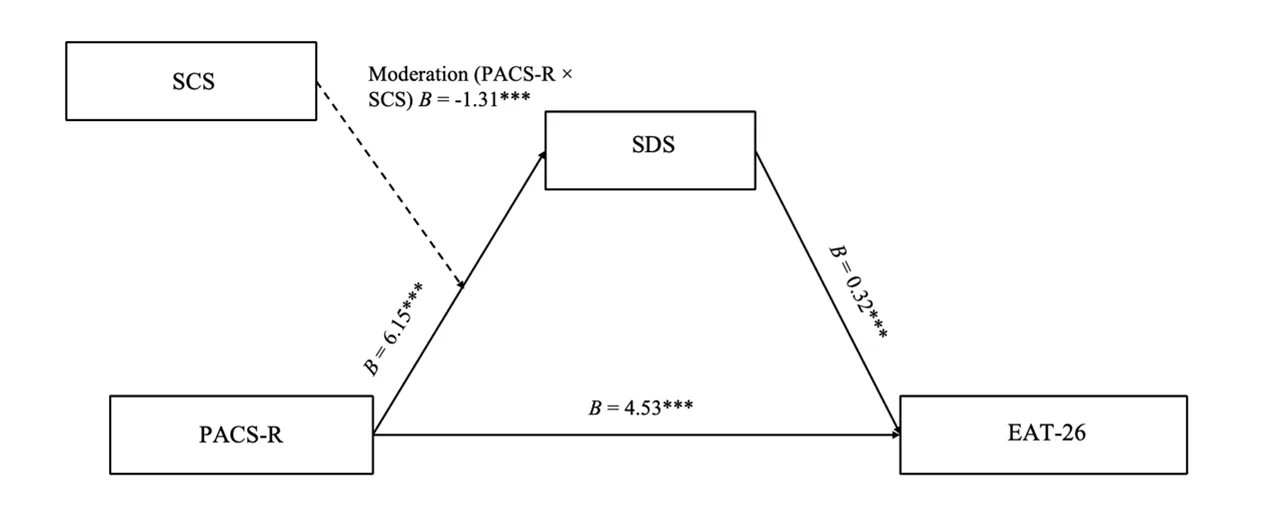
**Moderated mediation model of self-disgust (SDS) as a mediator between appearance-related comparisons (PACS-R) and disordered eating (EAT-26), with self-compassion (SCS) as a moderator**. Note. n = 812. The values represent unstandardized regression coefficients (B). The dashed line indicates the moderation effect on the first stage of the mediation (PACS-R × SCS). Sex was included as a covariate in all analyses. Significant paths are indicated with asterisks ***p < 0.001.

The model predicting disordered eating accounted for 36.7% of the variance. Using HC3 inference, appearance-related social comparison, B = 4.53, SEHC3 = 0.37, *p* < 0.001, and self-disgust, B = 0.32, SEHC3 = 0.05, *p* < 0.001, were both positively associated with disordered eating. The direct association between appearance-related social comparison and disordered eating remained significant after accounting for self-disgust, indicating that self-disgust explained part, but not all, of the association between these variables.

Conditional indirect effects indicated that the indirect association between appearance-related social comparison and disordered eating through self-disgust was significant at low, moderate, and high levels of self-compassion. However, the magnitude of this indirect effect decreased from 0.99 at low levels of self-compassion to 0.40 at high levels. The index of moderated mediation was significant (Index = −0.42, 95% BootCI [−0.69, −0.18]), indicating that the indirect association was weaker among individuals reporting higher self-compassion.

### Supplementary Analyses by Sex

To examine the generalizability of the proposed model across sexes, supplementary sex-moderated analyses were conducted. There was no evidence that sex moderated either the first-stage buffering effect of self-compassion on the association between appearance-related social comparison and self-disgust or the direct association between appearance-related social comparison and disordered eating. However, the association between self-disgust and disordered eating was significantly stronger among women than among men (B = 0.19, SE = 0.09, *p* = 0.035, 95% CI [0.01, 0.36]). Although the conditional indirect effect was significantly larger among women at low levels of self-compassion, the between-sex index of moderated mediation was not statistically significant (95% CI [−0.99, 0.02]). Full results are reported in **Supplementary Table 1**.

## Discussion

Appearance-related social comparison appears to become psychologically harmful when perceived appearance discrepancies are internalized as self-disgust rather than processed through more adaptive forms of self-relating. In the present study, self-disgust emerged as a mechanism linking appearance-based social comparison to disordered eating, whereas self-compassion weakened this pathway. Notably, the magnitude of the indirect association was reduced by more than half among individuals reporting higher levels of self-compassion. Together, these findings support the growing relevance of self-relational perspectives in eating psychopathology and suggest that vulnerability to disordered eating may depend not only on how individuals evaluate their appearance, but also on how they respond to themselves when confronted with perceived appearance inadequacies.

This perspective extends existing models of eating psychopathology in an important way. Traditional explanations have largely focused on body dissatisfaction, thin-ideal internalization, and self-objectification as mechanisms through which appearance-related experiences contribute to eating-related difficulties [1, 2, 3, 4]. While these factors remain highly relevant, they primarily address how individuals evaluate their appearance. Our findings indicate that understanding how individuals respond to those evaluations may be equally important. More specifically, appearance-based comparisons may confer vulnerability when perceived discrepancies are internalized as self-directed aversion and rejection. In this regard, self-disgust may represent a mechanism of internalization through which external appearance standards become translated into self-rejection and eating-related vulnerability.

This interpretation is consistent with the growing evidence identifying self-disgust as a clinically relevant construct in body image disturbance and eating disorders [9, 10, 12]. However, whereas previous research has primarily documented associations between self-disgust and eating-related difficulties, the present study suggests a more specific role for this construct. In particular, the findings indicate that self-disgust may help explain why appearance-related comparison constitutes a vulnerability factor for disordered eating, extending recent evidence positioning self-disgust as a central mechanism in the maintenance of negative body image [11, 13].

The most novel aspect of the present findings concerns the role of self-compassion. Previous research has consistently demonstrated that self-compassion is associated with lower levels of eating pathology and more positive body image [15, 16, 17, 31]. However, less is known about how this protective effect operates. Rather than acting solely as a correlate of lower eating pathology, self-compassion appeared to modify the process through which appearance-related experiences became psychologically harmful. Individuals high in self-compassion may still perceive appearance discrepancies and engage in appearance-based comparison, but they may be less likely to interpret those experiences as evidence of personal defectiveness. Consequently, appearance comparisons are less likely to be transformed into self-disgust. Notably, the magnitude of the indirect association between appearance-related social comparison and disordered eating was reduced by more than half among individuals reporting higher levels of self-compassion. This finding suggests that self-compassion may not eliminate physical appearance comparisons, but may substantially reduce their capacity to evolve into self-disgust and eating-related difficulties. Importantly, individuals high in self-compassion were not necessarily less exposed to appearance-related comparisons; rather, they appeared less likely to interpret those comparisons through a lens of self-rejection.

Importantly, self-compassion moderated the earliest stage of the proposed pathway. This finding suggests that self-compassion may not merely attenuate the consequences of self-disgust once it has emerged, but may reduce the likelihood that self-disgust develops in response to appearance-related challenges in the first place. In this sense, self-compassion appears to function as a resilience process that influences how appearance-related experiences are psychologically interpreted and integrated into the self-concept. This interpretation extends previous work showing that self-compassion buffers the association between self-disgust and drive for thinness [32] and highlights self-compassion as a potential mechanism of resilience within appearance-related contexts. More broadly, the findings support emerging perspectives suggesting that vulnerability to eating-related difficulties may depend not only on how individuals evaluate their appearance, but also on how they relate to themselves when confronted with perceived appearance inadequacies.

Clinical implications should be considered cautiously because the data are cross-sectional. Nevertheless, the findings suggest that assessment of self-disgust may provide information not captured by measures of body dissatisfaction alone and that interventions targeting hostile self-relating may complement established approaches. Compassion-focused interventions are relevant in this regard because they seek to reduce self-criticism and promote a more supportive relationship with the self [17, 33, 34]. Cognitive bias modification may offer another route for targeting maladaptive interpretations. A randomized trial of internet-based interpretation-bias modification found improvements in appearance-related interpretations and several body-image outcomes among individuals with elevated body dissatisfaction [35]. A small randomized trial in adolescents receiving treatment for eating disorders also reported improvements in interpretation bias and eating-disorder symptoms when attention and interpretation training was added to usual care [36]. These findings are preliminary and do not establish that modifying interpretation bias would reduce self-disgust specifically; this possibility requires direct testing.

The study has several strengths, including its large sample size, the use of psychometrically robust measures, and the simultaneous examination of vulnerability and protective processes within a single model. A further strength lies in the examination of maladaptive and adaptive self-relational processes within the same explanatory framework.

Nevertheless, several limitations should be acknowledged. The cross-sectional design precludes causal conclusions and alternative temporal orderings remain plausible. For example, disordered eating may contribute to greater physical appearance comparison and self-disgust over time. In addition, all variables were assessed through self-report measures, and the use of a non-probabilistic community sample limits generalizability. Future longitudinal and clinical studies should examine whether self-disgust prospectively predicts eating pathology and whether reductions in self-disgust represent a mechanism through which self-compassion-based interventions exert their effects.

## Conclusions 

The present study suggests that appearance-related social comparison may contribute to disordered eating through its association with self-disgust. More importantly, the findings indicate that self-compassion weakens this process, reducing the likelihood that appearance-related discrepancies become internalized as self-directed aversion. These results support the growing relevance of self-relational perspectives in eating psychopathology and suggest that vulnerability to eating-related difficulties may arise not only from how individuals evaluate their appearance, but also from how they relate to themselves when confronted with perceived appearance inadequacies. Understanding and modifying these self-relational processes may therefore represent an important avenue for the prevention and treatment of disordered eating. Taken together, the findings suggest that the consequences of appearance-related comparison may depend less on the comparison itself than on how individuals relate to themselves in response to perceived appearance discrepancies.

## Availability of Data and Materials

The datasets used and/or analyzed during the current study are available from the corresponding author on reasonable request.
