# Supplementary material for: When Appearance Comparisons Become Self-Disgust: The Protective Role of Self-Compassion in Disordered Eating
Source: Actas Esp Psiquiatr. 2026 Oct 15;54(5):1468–77. doi: 10.62641/aep.2439 (PMC13714456; doi:10.62641/aep.2439)
Supplement: Supplementary file 1 [file ActEsp-54-5-1468-1477-s1.zip › AEP-2439-Supplementary materials-Final version.docx]

**Supplementary Materials**

**Table 1.** Supplementary tests of sex differences in the moderated mediation pathways

| ***Effect*** | ***Estimate*** | ***SE/BootSE*** | ***p*** | ***95% CI*** |
| --- | --- | --- | --- | --- |
| PACS-R × sex → self-disgust | 0.23 | 0.60 | 0.706 | [−0.96, 1.41] |
| PACS-R × SCS × sex → self-disgust | −0.92 | 0.80 | 0.250 | [−2.49, 0.65] |
| PACS-R × sex → disordered eating | −0.25 | 0.74 | 0.732 | [−1.71, 1.20] |
| Self-disgust × sex → disordered eating | 0.19 | 0.09 | 0.035 | [0.01, 0.36] |
| Men: indirect effect at low SCS | 0.52 | 0.19 |  | [0.20, 0.97] |
| Men: indirect effect at median SCS | 0.44 | 0.16 |  | [0.19, 0.80] |
| Men: indirect effect at high SCS | 0.35 | 0.17 |  | [0.06, 0.74] |
| Women: indirect effect at low SCS | 1.31 | 0.30 |  | [0.79, 1.99] |
| Women: indirect effect at median SCS | 0.92 | 0.24 |  | [0.52, 1.47] |
| Women: indirect effect at high SCS | 0.49 | 0.25 |  | [0.05, 1.03] |
| Women − men: indirect effect at low SCS | 0.80 | 0.36 |  | [0.12, 1.55] |
| Women − men: indirect effect at median SCS | 0.49 | 0.28 |  | [−0.04, 1.07] |
| Women − men: indirect effect at high SCS | 0.14 | 0.30 |  | [−0.43, 0.77] |
| Women − men: index of moderated mediation | −0.47 | 0.26 |  | [−0.99, 0.02] |

*Note. PACS-R = Physical Appearance Comparison Scale–Revised; SCS = Self-Compassion Scale; SE = standard error; BootSE = bootstrap standard error; CI = confidence interval; HC3 = heteroscedasticity-consistent estimator. Sex was coded 0 = men and 1 = women. Regression SEs are HC3 estimates. Indirect effects and between-sex differences were estimated using 5,000 percentile bootstrap samples; their significance was determined from 95% bootstrap CIs, so p values are not reported. Low, median, and high SCS denote the 16th, 50th, and 84th percentiles, respectively.*
